# Optical, Electrical, Structural, and Thermo-Mechanical Properties of Undoped and Tungsten-Doped Vanadium Dioxide Thin Films

**DOI:** 10.3390/ma17102382

**Published:** 2024-05-16

**Authors:** Chuen-Lin Tien, Chun-Yu Chiang, Ching-Chiun Wang, Shih-Chin Lin

**Affiliations:** 1 Department of Electrical Engineering, Feng Chia University, Taichung 40724, Taiwan; 2 Ph.D. Program of Electrical and Communications Engineering, Feng Chia University, Taichung 40724, Taiwan; hank4681898@gmail.com; 3 Mechanical and Systems Research Lab, Industrial Technology Research Institute, Hsinchu 310401, Taiwan; juin0306@itri.org.tw (C.-C.W.); shihchin@itri.org.tw (S.-C.L.)

**Keywords:** vanadium dioxide, thin film, electron beam evaporation, ion-assisted deposition

## Abstract

The undoped and tungsten (W)-doped vanadium dioxide (VO_2_) thin films were prepared by electron beam evaporation associated with ion-beam-assisted deposition (IAD). The influence of different W-doped contents (3–5%) on the electrical, optical, structural, and thermo-mechanical properties of VO_2_ thin films was investigated experimentally. Spectral transmittance results showed that with the increase in W-doped contents, the transmittance in the visible light range (400–750 nm) decreases from 60.2% to 53.9%, and the transmittance in the infrared wavelength range (2.5 μm to 5.5 μm) drops from 55.8% to 15.4%. As the W-doped content increases, the residual stress in the VO_2_ thin film decreases from −0.276 GPa to −0.238 GPa, but the surface roughness increases. For temperature-dependent spectroscopic measurements, heating the VO_2_ thin films from 30 °C to 100 °C showed the most significant change in transmittance for the 5% W-doped VO_2_ thin film. When the heating temperature exceeds 55 °C, the optical transmittance drops significantly, and the visible light transmittance drops by about 11%. Finally, X-ray diffraction (XRD) and scanning electron microscope (SEM) were used to evaluate the microstructure characteristics of VO_2_ thin films.

## 1. Introduction

Vanadium oxide is a typical thermochromic material exhibiting a metal-insulator transition (MIT) near room temperature. Unprecedented modulation of IR transmission is achieved by the addition of up-conversion nanoparticles [1]. Vanadium can exist in various oxidation states, forming different oxides, and obtaining high-purity vanadium oxide presents challenges, requiring optimal temperature and pressure conditions. Vanadium dioxide (VO_2_) has garnered widespread attention due to its unique insulator-to-metal phase transition characteristics. As the temperature changes, VO_2_ undergoes a reversible transition between the insulating and metallic states, leading to significant changes in its electrical and optical properties. For undoped VO_2_, the phase transition temperature is typically 68 °C, limiting its application in various energy-saving-related uses that require room temperature as the control threshold [2]. VO_2_ enables the transition from insulator to metal (IMT), and the VO_2_ transition from a monoclinic (M1) to a rutile structure (R) occurs around 68 °C. At temperatures below 68 °C, a monoclinic VO_2_ is stable and demonstrates semiconducting behaviors. Meanwhile, at temperatures above 68 °C, rutile VO_2_ is stable and exhibits metallic characteristics that result in infrared reflection [3]. Different metallic vanadium thin film surface roughness and grain sizes have different oxidation times to make VO_2_ thin film. Oxidation time can deeply affect phase transition properties and the crystallization of VO_2_ thin film [4]. Thus, VO_2_ serves as a potential thermochromic material for smart windows that regulates infrared transmittance in response to changes in temperature.

In recent years, VO_2_ has received widespread attention due to its significant reversible changes in electrical and optical properties, especially near 68 °C, making it more suitable for practical technical applications. However, the transition temperature (Tt) of VO_2_ is still too high for energy applications related to room temperature [5]. In most cases, reducing the Tt to near ambient levels is essential for optimizing the advantages of variable solar energy influx. This goal has driven research efforts to lower Tt through the doping metal ions. Dou et al. [6] reported that tungsten doping is feasible to reduce the phase transition temperature. W-doped VO_2_ films offer several advantages, including reducing the phase transition temperature, narrowing the width of hysteresis loops, and improving cycle stability. For W-doped VO_2_, the decrease in the transition temperature is attributed to the formation of V^3+^–W^6+^ pairs through the crystallization of VO_2_ into a monoclinic structure, resulting in a weakening of the bonding strength between V ions. The formation of these V^3+^–W^6+^ pairs occurs as two electrons from the d-orbital of tungsten move to adjacent V ions for charge compensation. Thus, with an increase in the W^6+^ content in the lattice, the V^4+^–V^4+^ bond weakens, leading to semiconductor phase instability and a decrease in the transition temperature. The synthesized W-doped VO_2_ thin film presents Tt = 47 °C [7]. Takami et al. [8] experimentally found that tungsten doping increases carrier concentration, altering the Coulomb repulsion energy and stabilizing the metallic state of the thin film. However, the growth of tungsten-doped vanadium dioxide (W:VO_2_) also presents challenges, such as the instability of the ability to lower the phase transition temperature of VO_2_ through tungsten doping, with significant variations in results based on different preparation techniques, especially in achieving precise tungsten doping over large surface areas. Haining [9] et al. prepared a thin film that lowers the phase transition temperature of vanadium dioxide by adjusting the doping ratio of tungsten. When the tungsten doping concentration is set to 1.70%, the phase transition temperature of the VO_2_ thin film is reduced from 67 °C to 43.2 °C. It is worth mentioning that the infrared characteristics of tungsten-doped vanadium dioxide nanoparticles were investigated in mid-wave infrared (3–5 μm) and long-wave infrared (8–14 μm) bands. They showed an excellent solar light modulation rate (ΔT_sol_) of 23%, which is accompanied by the modulation of emissivity. Their findings further advance the potential application and development of smart windows. Ye and Zhou [10] proposed that when tungsten is successfully doped into the vanadium dioxide lattice, the tungsten-doped vanadium dioxide will exhibit a rod-like morphology, and they discussed the phase transition temperature and annealing temperature. The phase transition temperature of vanadium dioxide can be adjusted by changing the doping concentration of tungsten. When the doping concentration is 1.58 mol %, the phase transition temperature can be reduced from the initial 69.5 °C to 37.8 °C, indicating that tungsten doping vanadium dioxide can exhibit outstanding thermochromic properties.

This work aims to investigate the effect of W-doped contents on the optical, electrical, structural, and thermo-mechanical properties of the thermochromic vanadium dioxide thin films. Understanding the properties of VO_2_ thin films from various instrument measurements can help improve the film’s durability for applications in smart windows, optoelectronic switches, and intelligent heat dissipation devices.

## 2. Materials and Methods

### 2.1. Preparation of Tungsten-Doped Vanadium Dioxide Thin Films with Different Contents

Electron beam evaporation is a technique capable of achieving the growth of thin films over large surface areas. The evaporation chambers are typically configured to accommodate multiple substrates simultaneously, thus demonstrating exceptional production efficiency. This method offers advantages such as reduced processing time and ease of maintenance, rendering it a prevalent choice in industrial applications for producing optical coatings, transparent conductive films, and related products [11,12]. This study utilized the SHOWA ion-assisted electron beam deposition system to deposit tungsten-doped vanadium oxide thin films with different doping contents. Tungsten doping concentrations of 0%, 3%, 4%, and 5% were used in the process. B270 and H-K9L glass substrates with a diameter of 25 mm were used, and the substrate heating temperature was maintained at 240 °C throughout the process. The electron beam generated by the electron gun bombarded the material and deposited it onto the substrate. The high vacuum coating system consists of a mechanical booster pump and an oil rotary pump (RP) to facilitate the initial evacuation of the vacuum chamber and ensure optimal vacuum conditions for subsequent processes. This evacuation operation process improves the efficiency of the vacuum system by effectively capturing water vapor, thereby maintaining the vacuum level required for various applications and increasing the efficiency of the evacuation rate. In this work, the VO_2_ thin films were produced through electron beam evaporation and ion-beam-assisted deposition techniques. To maintain ideal conditions for the deposition process and achieve a controlled environment for high-quality film formation. We depressurized the high vacuum chamber to a base pressure below 2.7 × 10^−4^ Pa. This low-pressure environment is crucial for achieving precise deposition and optimizing the quality of the resulting thin films. The VO_2_ thin films were then deposited on various substrates, including B270 and H-K9L glass substrates with a diameter of 25 mm, as well as on silicon wafer substrates, to study the thin film’s properties on different materials and support versatile measurements and applications. These substrates designated for coating are carefully cleaned using ultrasonic waves to remove any contaminants. During the coating process, high-quality thin film coatings and optimal deposition conditions were achieved. The purity of 99.999% argon and oxygen are used during the deposition process. During the deposition of the VO_2_ thin films, the electron gun’s peak power output was set at 10 kW to guarantee effective and well-regulated film formation. A voltage of 10 kV and a current of 1 A were set as coating parameters. For the ion source utilized in ion-assisted deposition, the anode current ranged from 0.5 to 10 A, the anode voltage varied from 80 to 300 V, and the ion energy spanned 50 to 200 eV. The measurement of film thickness is achieved through a hybrid approach that combines both quartz crystal and optical monitoring techniques. The optical monitoring apparatus utilizes a spectrometer capable of measuring wavelengths from 360 nm to 1000 nm, while the quartz crystal monitoring setup employs a 5 MHz quartz crystal oscillator to facilitate precise and accurate quantification of film thickness. The hybrid monitoring approach ensured precise and accurate measurements of film thickness during the deposition process. The optical system detected changes in reflectance during the thin film coated onto glass substrates and used the extrema of reflectance to control the deposition process. In this study, the deposition thickness of all VO_2_ thin films was uniformly set to 100 nm. The vacuum pressure throughout the coating process was held consistently below 6 × 10^−4^ Pa, ensuring controlled deposition conditions. The power of the electron beam for VO_2_ evaporation was also maintained at 7.5 kW. The working pressure was 3.0 × 10^−2^ Pa. The deposition rate was 0.1 nm/s. The oxygen gas injected during the VO_2_ layer deposition was 20 sccm. The ion-assisted deposition process involved an argon flow rate of 16 sccm for the VO_2_ layer. The anode voltage and anode current used for thin film deposition were set at 130 V and 2 A, respectively. During the VO_2_ thin film deposition process, the heating temperature of the various substrates was elevated to 280 °C.

### 2.2. Characterization Measurement of Tungsten-Doped Vanadium Dioxide Thin Films

To understand the post-deposition characteristics of the VO_2_ thin films, this research employed a UV-VIS-NIR spectrophotometer (Shimadzu UV-2600i) and a temperature-controlled heating stage. Spectral measurements were performed by two instruments: one is a UV-VIS-NIR spectrophotometer covering the wavelength range from 200 nm to 1000 nm, and transmittance measured after heating. The other is Fourier transform infrared spectroscopy (FTIR), which measures transmittance in the infrared range of 2.5 μm to 5.5 μm. The infrared transmittance of VO_2_ thin films at normal incidence was evaluated using Fourier transform infrared spectrometer (Thermo Fisher Scientific, Waltham, MA, USA). A homemade Twyman–Green interferometer and Linnik microscopic interferometer were utilized to measure the residual stress and root-mean-square surface roughness of the VO_2_ thin films [13,14]. Additionally, Raman spectroscopy analysis was employed to examine the crystal structure of the thin films.

The residual stress in the thin film was evaluated using a homemade Twyman–Green interferometer. The optical interferometer is used according to the method described in the author’s previous publications [13]. The He–Ne laser beam (wavelength 632.8 nm) is focused into a point source through a microscope objective and pinhole, which also act as a spatial filter. The laser beam then passes through a collimating lens to produce a plane wavefront. The wavefront is split in amplitude by a beam splitter, resulting in two beams: one reflected toward a reference mirror (with a flatness of λ/20) and the other transmitted toward the glass substrate under test. A glass substrate polished on one side is placed on a three-axis platform to introduce spatial carrier frequency and produce interference fringes. After being reflected by the reference mirror and glass substrate, the beam is recombined through a splitter and directed toward a 1920 × 1200 pixel digital CMOS camera (Basler, Littleton, CO, USA). The interference pattern is displayed on a monitor connected to the CMOS camera. The interferogram is captured and processed by a personal computer utilizing a custom MATLAB program (R2023b, MathWorks, Natick, MA, USA) designed for comprehensive thin film stress analysis.

For the surface roughness measurement, a homemade surface roughness measuring system based on the Linnik microscopic interferometer was employed to determine the surface roughness of the thin films deposited on the glass substrate [14]. This system consisted of a Linnik-type interference microscope outfitted with two 50× microscope objective lenses, a two-axis translation stage, and a CCD camera for imaging the surface of the specimen under investigation. A custom MATLAB program, leveraging FFT algorithms, was employed to analyze the captured interference images for detailed characterization. A He-Ne laser is used as the light source to emit laser beams. The laser beam passes through a spatial filter and a collimating lens to form a parallel beam. The incident light is then divided by a beam splitter into two parallel beams. One beam passes through a 50× microscope objective lens to be reflected by a reference mirror; the other beam strikes the surface of the test sample after passing through a 50× microscope objective lens. When two reflected beams are recombined by the beam splitter, an interference pattern is generated on the image plane. The resulting interference fringes are then captured using a high-resolution CCD camera, and then the interferograms are processed by using a self-developed MATLAB program. The analytical program predominantly relies on the fast Fourier transform (FFT) method to detect the height differences for the thin film surface. In addition, a digital Gaussian filter is employed to define the signal cut-off wavelength, thereby separating high-frequency roughness components from the low-frequency surface profile. This methodology facilitates the reconstruction of the three-dimensional surface profile of the thin film, and the surface roughness parameters are determined by numerical analysis [15]. The electrical property of the VO_2_ thin film was tested with the standard four point probe method. In addition, X-ray diffraction (XRD), using a SIEMENS D-5000 diffractometer (Siemens, Munich, Germany), is a powerful analytical method for characterizing materials and understanding their structural features. The microstructure of VO_2_ thin films was analyzed using a Hitachi S-4800 field emission scanning electron microscope (FE-SEM, Hitachi High-Tech, Tokyo, Japan).

## 3. Results and Discussion

### 3.1. Optical Properties of Undoped VO_2_ and W-Doped VO_2_ Thin Films

#### 3.1.1. Transmission Spectral Characteristics of Undoped VO_2_ and W-Doped VO_2_ Thin Films

**Figure 1:**
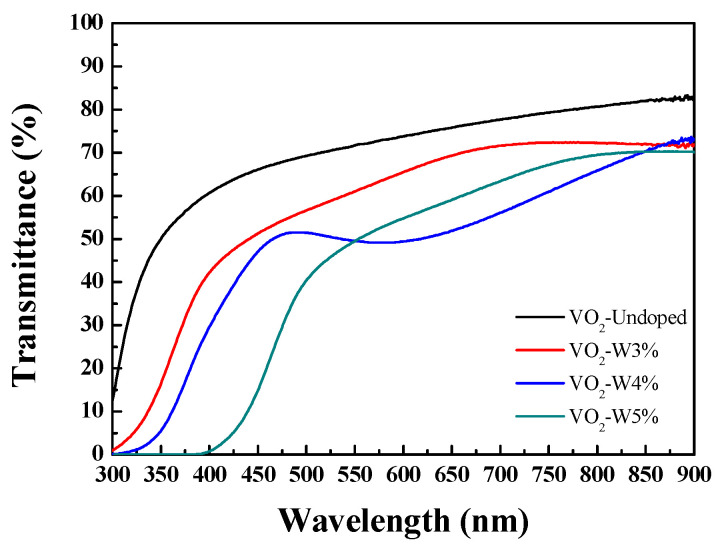
Transmittance spectra of undoped and W-doped VO_2_ thin films.

Optical spectral characteristics of W-doped VO_2_ thin films were measured with the Shimadzu UV-2600i UV-Visible spectrophotometer (Shimadzu Corporation, Kyoto, Japan). From the UV-visible light measurement results, the average transmittance of B270 glass substrate coated with W-doped VO_2_ thin films with different contents of 3%, 4%, 5%, and undoped VO_2_ thin film was determined in the wavelength range of 300–900 nm, as shown in Figure 1. The visible light transmittances of 3%, 4%, 5%, and undoped VO_2_ thin films are 60.2%, 56.3%, 53.9%, and 67.2%, respectively. Tungsten doping contents can influence the crystal structure of vanadium dioxide, such as grain size and grain boundary characteristics. As the doping concentration increases, the grain size of the VO_2_ thin film gradually increases. These changes increase light scattering and reflection, thereby affecting optical transmittance.

#### 3.1.2. Infrared Transmittance Spectra of Undoped VO_2_ and W-Doped VO_2_ Thin Films

**Figure 2:**
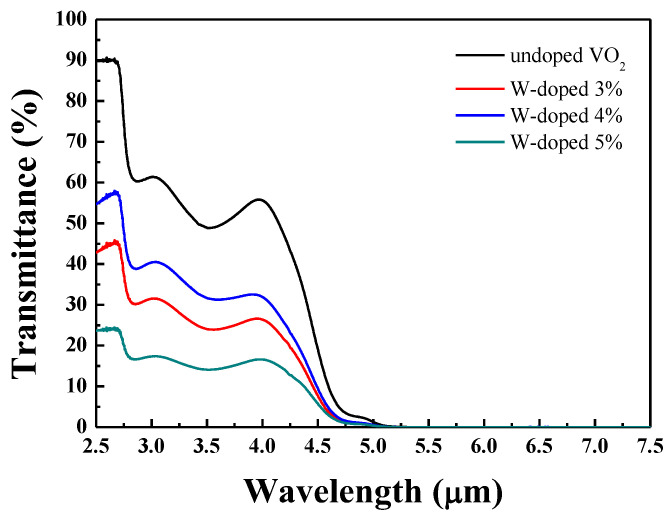
Infrared transmittance spectra of undoped and W-doped VO_2_ thin films.

Fourier transform infrared spectroscopy (FTIR) is used to determine the infrared transmittance. The transmittance spectra of VO_2_ thin films are obtained by varying the tungsten dopant concentration in the wavelength range of 2.5 μm to 5.5 μm. The average transmittance values of 3%, 4%, 5%, and undoped VO_2_ thin films are 55.8%, 32.5%, 26.5%, and 15.4%, respectively, as shown in Figure 2. As the W doping concentration increases, the infrared transmittance decreases. Tungsten is a dense material that exhibits high absorption and scattering in the infrared wavelength range due to its electronic structure and properties. The doping of tungsten changes the crystal structure of vanadium dioxide, causing the grain size to increase as the tungsten doping concentration gradually increases. This also results in increased light scattering and reflectance, which affects its transmittance.

#### 3.1.3. Refractive Index of Undoped VO_2_ and W-Doped VO_2_ Thin Films

**Figure 3:**
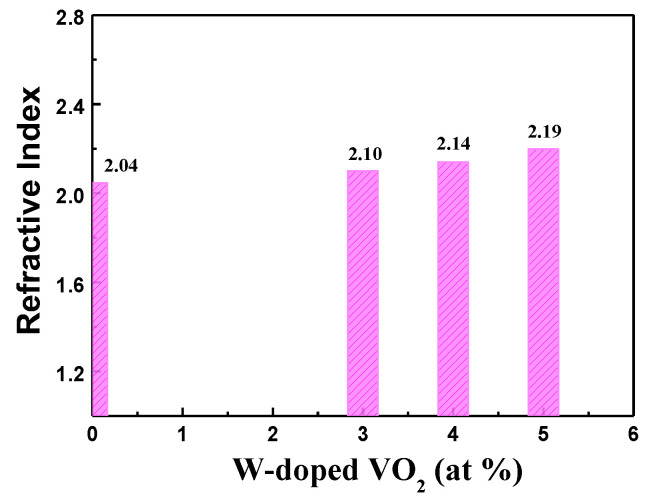
The refractive index of VO_2_ thin films with different W-doped contents.

The optical refractive index (n), extinction coefficient (k), and film thickness of undoped and W-doped VO_2_ thin films were measured using an ellipsometer. A spectroscopic ellipsometer is a commonly utilized instrument for the analysis and measurement of thin films. The spectroscopic ellipsometer combines rotational analyzer ellipsometer technology to characterize thin film samples. Spectral ellipsometry measures the change in polarization of light after it reflects from a sample. The detector of an ellipsometer measures the quantities Ψ and Δ at each corresponding wavelength or photon energy. The parameter Ψ represents the ratio of the amplitudes of p-polarized and s-polarized reflected light, and Δ represents their phase difference. Generally, the electric field vector of p-polarized light is parallel to the incident plane, while the electric field vector of s-polarized light is perpendicular to the incident plane. The spectroscopic ellipsometer can precisely determine optical constants, including film thickness, refractive index, and extinction coefficient. Figure 3 shows the refractive index of undoped and W-doped VO_2_ thin films. In the Figure 3, it can be seen that the refractive index of VO_2_ thin films increases with the increase in tungsten doping concentration. The refractive index value slightly increases, but the extinction coefficient shows a relatively small change. Tungsten is a metal with a higher atomic number, which has a larger atomic size and mass. When tungsten is doped into VO_2_ thin films, its larger atomic mass increases the average atomic mass of the film, thereby improving material density and atomic arrangement. This reason leads to an increase in refractive index.

#### 3.1.4. Raman Spectra of W-Doped VO_2_ Thin Films

**Figure 4:**
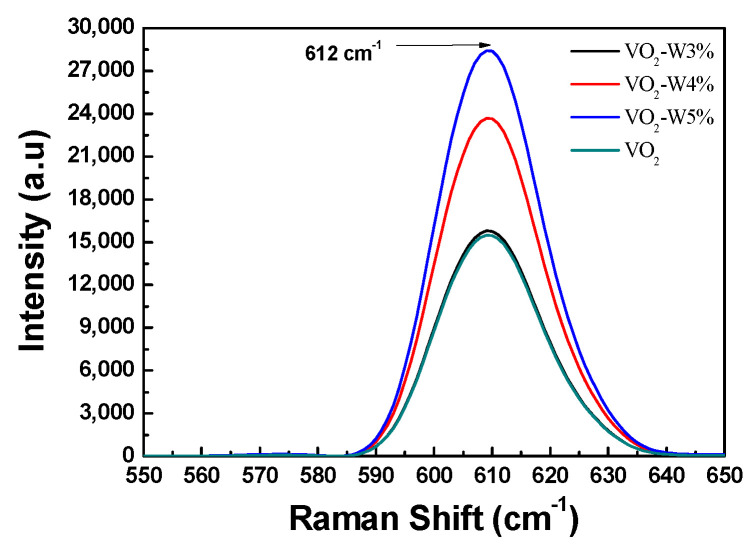
Raman spectra of undoped and tungsten-doped vanadium dioxide thin films.

Raman spectroscopy is a form of vibrational spectroscopy, like infrared (IR) absorption, that provides detailed chemical and structural characterization. We performed room-temperature Raman spectroscopy measurements of the four VO_2_ thin films, with laser excitation at 785 nm. Figure 4 indicates the Raman spectroscopy for the measurements of four VO_2_ thin films. In the case of different W doping contents, the Raman spectra of undoped and W-doped VO_2_ thin films were measured. Figure 4 shows the characteristic peak for different doping contents (undoped 0%, 3%, 4%, and 5%) was located at 612 cm^−1^, with intensities of approximately 14,320 (a.u), 15,650 (a.u), 24,680 (a.u), and 29,860 (a.u), respectively. Among the W-doped samples, the 5% W-doped VO_2_ thin film exhibited the highest peak intensity, indicating a better crystallinity of the film compared to the other samples. The characteristic peak signal of vanadium oxide is located at 612 cm^−1^. No discernible features correspond to other vanadium oxides [16]. From Figure 4, it can be observed that the detected peak values for the undoped and three different W doping samples match those reported in the literature [17]. The Raman peak position corresponds to the mode of the VO_2_ monoclinic (M) phase. In addition, from the Raman spectra of W-doped VO_2_ thin films, it can be seen that compared with the undoped VO_2_ thin film, these peaks are significantly stronger and have a wider peak width. These results confirm that the substitution of W atoms in the VO_2_ lattice induces local rutile structure, and then the original semiconductor phase of VO_2_ exhibits partial metallic behavior [18]. Furthermore, we observed that the peak broadens as the W doping percentage increases. This can be explained as W doping beginning to favor a more symmetrical rutile structure. A similar effect on W-doped VO_2_ thin films has already been reported in the literature [19,20].

#### 3.1.5. Temperature-Dependent Transmission Spectra of VO_2_ Thin Films

**Figure 5:**
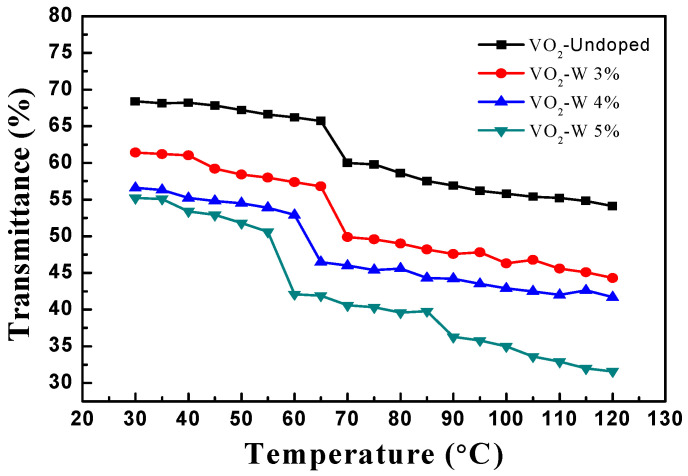
Transmittance of undoped and W-doped VO_2_ thin films as a function of heating temperatures.

The temperature-dependent properties of VO_2_ thin films are key characteristics for the application of thermochromic materials [21]. For example, temperature-dependent optical transmittance measurement shows the large switching efficiency of VO_2_ (M) thin films at the phase transition temperature (Tt) around 64.28 °C. In W-doped VO_2_ thin films, a decrease in phase transition temperature can be observed, which is due to the change in the electronic structure of VO_2_ film caused by doping [22]. In general, the Tt reduction in VO_2_ thin films is caused by several parameters, such as residual stresses, film thickness, stoichiometry, etc., which are directly related to the selected processing conditions [23,24,25]. The understanding of the annealing time-dependent modulation in properties of VO_2_ thin films can help to stabilize VO_2_ with desired functional properties [26]. In this study, the undoped and W-doped VO_2_ thin films were heated from 30 °C to 100 °C using a heating stage. As the heating temperature increases, the sample’s temperature measurements are taken every 5 °C interval. Optical transmission spectral signals were measured using a spectrophotometer. We employed the W-doped contents of 0%, 3%, 4%, and 5% as VO_2_ thin film samples. From the optical transmittance measurement data, it can be observed that as the heating temperature rises, the optical transmittance values of all vanadium dioxide films with different doping contents gradually decrease, as shown in Figure 5. This phenomenon can be attributed to the impact of elevated temperature on the internal lattice structure, atomic spacing, and electronic band structure of the thin films, resulting in changes in the refractive index and lattice scattering within the thin films. The transmission spectra of VO_2_ thin films with different W doping concentrations indicate that the transmittance of VO_2_ thin films decreases with the increase in W doping concentration. It can be observed that when the VO_2_ thin film sample is heated above 55 °C, the transmittance significantly decreases. Compared to the lower temperature of 30 °C, the transmittance in the visible light wavelength range is reduced by approximately 11%. It is speculated that the phase transition occurs around 68 °C, with VO_2_ transforming into a metallic phase [27], resulting in a significant reduction in transmittance. The transmittance of VO_2_ thin films with a W doping concentration of 5% significantly decreases from 55 °C, while the undoped VO_2_ thin films show significant changes after 65 °C.

### 3.2. Thermo-Mechanical Properties of Undoped VO_2_ and W-Doped VO_2_ Thin Films

#### 3.2.1. Residual Stress of Undoped VO_2_ and W-Doped VO_2_ Thin Films

**Figure 6:**
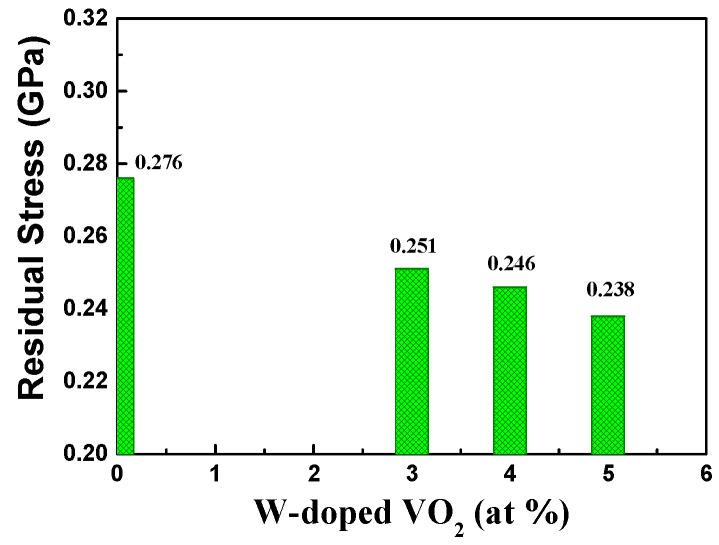
Residual stress of undoped VO_2_ and W-doped VO_2_ thin films.

The measurement of residual stress was evaluated by a homemade Twyman–Green interferometer. The measuring results show the residual stress in a compressive stress state. The residual stresses of VO_2_ thin films with tungsten doping contents of 3%, 4%, 5%, and undoped VO_2_ are −0.251 GPa, −0.246 GPa, −0.238 GPa, and −0.276 GPa, respectively, as shown in Figure 6. An increase in doping concentration leads to a reduction in residual stress in the films. The doping concentration of tungsten influences the phase transition temperature of vanadium oxide thin films, and changes in the phase transition temperature can induce stress generation or alteration in the material within a specific temperature range. With the increase in tungsten doping concentration, the addition of dopant atoms can lead to lattice modulation or alter the crystalline structure of the film. These changes contribute to alleviating internal stress in the film, thereby reducing the level of residual stress.

#### 3.2.2. Surface Roughness Measurement of Undoped VO_2_ and W-Doped VO_2_ Thin Films

**Figure 7:**
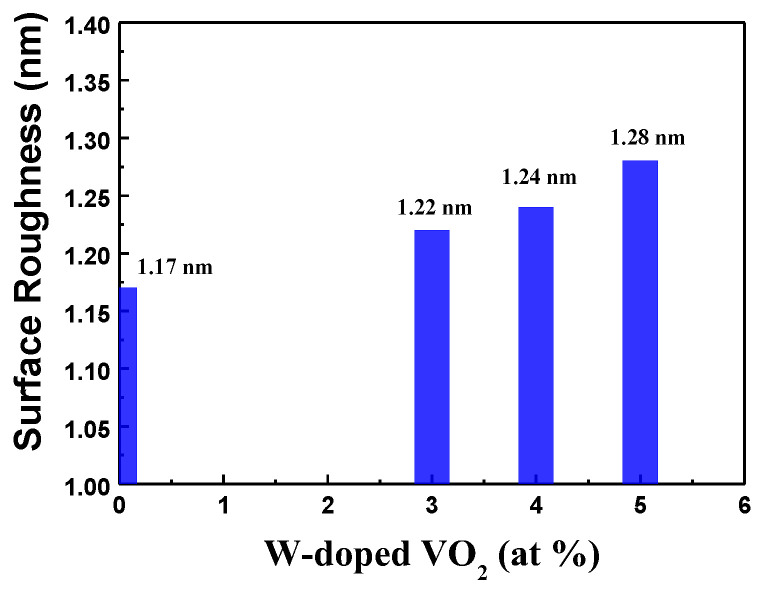
Surface roughness of undoped VO_2_ and W-doped VO_2_ thin films deposited on B270 glass substrates.

The root mean square (RMS) surface roughness of VO_2_ films deposited on B270 glass substrates was evaluated by using a Linnik micro interferometer, and interference image processing and analysis were performed. Figure 7 shows the surface roughness measurement results of tungsten doped with concentrations of 3%, 4%, and 5%, as well as undoped vanadium dioxide films. The RMS surface roughness values of 3%, 4%, 5%, and undoped VO_2_ films are 1.17 nm, 1.22 nm, 1.24 nm, and 1.28 nm, respectively. It is evident that the surface roughness gradually increases as the tungsten doping concentration increases. An increase in tungsten concentration causes the surface morphology of the thin film to become rougher. This is explained as that the radius of the W^6+^ ion (0.060 nm) is slightly larger than that of the V^4+^ ion (0.058 nm). Higher tungsten concentrations cause uneven distribution of dopant atoms or generate internal stresses in the film structure, leading to surface irregularities and, thus, increased surface roughness.

#### 3.2.3. Temperature-Dependent Residual Stress of W-Doped VO_2_ Thin Films

**Figure 8:**
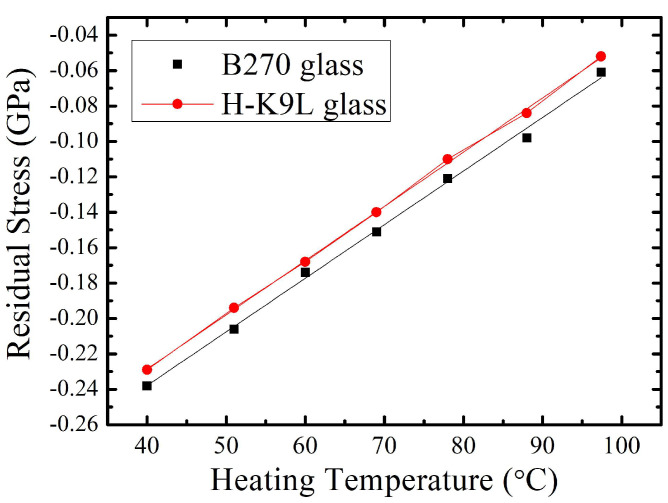
Residual stress as a function of heating temperature for 5% W-doped VO_2_ thin films deposited on dual substrates.

In this work, 5% W-doped vanadium dioxide thin films were simultaneously deposited on B270 glass and H-K9L glass substrates. The residual stress of the films was measured at temperature intervals of 10 °C from 30 °C to 100 °C. The VO_2_ thin films were coated on both B270 glass and H-K9L glass substrates to assess the residual stress–temperature variation relationship and obtain their gradient values, as shown in Figure 8. The calculation of thin film thermal stress involves determining the thermal expansion coefficient and biaxial modulus of the film through the residual stress–temperature variation curve. The thermal expansion coefficient and biaxial modulus of the thin film are obtained by calculations using Equations (1) and (2) [28,29].

(1) $$\alpha_{f}=\frac{\alpha_{1}\frac{d\sigma_{2}}{dT}-\alpha_{2}\frac{d\sigma_{1}}{dT}}{\frac{d\sigma_{2}}{dT}-\frac{d\sigma_{1}}{dT}}$$

E_f_/(1 − ν_f_) = ((ⅆσ_2_)/ⅆT − (ⅆσ_1_)/ⅆT)/(α_2_ − α_1_) (2)

where *α*_1_ and *α*_2_ represent the thermal expansion coefficients of the different glass substrates, the thermal expansion coefficients are 8.2 × 10^−6^ °C^−1^ for B270 glass and 7.6 × 10^−6^ °C^−1^ for H-K9L glass substrate, *α_f_* is the thermal expansion coefficient of the thin film to be determined, and *E_f_* and *ν_f_* denote the Young’s modulus and Poisson’s ratio of the thin film material, respectively. Here, $\frac{d\sigma_{1}}{dT}$ and $\frac{d\sigma_{2}}{dT}$ represent the slope values of the stress–temperature curves for the thin film deposited on the two different substrates.

For the 5% tungsten-doped vanadium oxide thin films with better performance, a dual substrate method was conducted using B270 and HK9L glass substrates. From the stress–temperature slope graphs for each temperature and residual stress on both substrates, slight fluctuations were observed. It is speculated that the fluctuations are caused by internal structural changes, and it cannot be entirely attributed to thermal stress. The growth distribution of thermal stress appears to be linear. By using Equations (1) and (2), the thermal expansion coefficient of the vanadium dioxide thin film was determined to be 5.1 × 10^−6^ °C^−1^, and the biaxial modulus was found to be 396 GPa. Based on the residual stress–temperature measurement results, it is inferred that the thermal stress of B270 and HK9L glass substrates increases linearly from 30 °C to 100 °C. Thermal stress occurs due to the thermal expansion coefficient mismatch between the film and the coating substrate, resulting in changes in surface morphology. It is worth noting that the thermal stress of the VO_2_ thin film remains relatively stable around 60–70 °C but exhibits linear growth. The stress–temperature variation trend of W-doped VO_2_ thin films needs further research to explore the actual changes in the internal structure of VO_2_ thin films.

#### 3.2.4. Temperature-Dependent Electrical Resistivity of Undoped VO_2_ and W-Doped VO_2_ Thin Films

**Figure 9:**
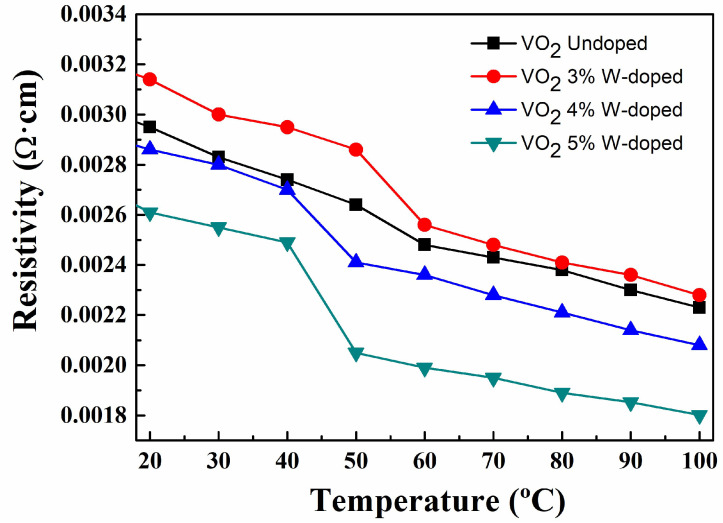
Temperature-dependent resistivity of undoped VO_2_ and W-doped VO_2_ thin Films.

The electrical properties of the undoped VO_2_ and W-doped VO_2_ thin films were measured using a commercial four-point probe. During the measurements, the VO_2_ thin film was placed on a temperature control stage, and its temperature is adjusted from 30 °C to 100 °C at intervals of 10 °C. Figure 9 shows the temperature-dependent resistivity measured with different tungsten doping contents and undoped VO_2_ thin films. It can be observed from Figure 9 that as the heating temperature of the VO_2_ thin film increases, the resistivity of the VO_2_ thin film gradually decreases with W-doped contents. Among them, the resistivity change of the VO_2_ films with 5% W-doped is the most significant. When the heating temperature increases from 20 °C to 100 °C, the resistivity of 5% W-doped VO_2_ thin film decreases from 2.61 × 10^−3^ (Ω·cm) to 1.80 × 10^−3^ (Ω·cm). This could be attributed to the carrier density increase with the temperature. VO_2_ thin film undergoes an insulator-to-metal phase transition within a specific temperature range. At low temperatures, vanadium dioxide is an insulator, but when the heating temperature is increased to a certain critical temperature, it transforms into a metallic state. In the metallic state, the mobility of electrons increases, resulting in an increase in conductivity and therefore a decrease in resistivity.

### 3.3. Microstructural Properties of VO_2_ Thin Films

#### 3.3.1. X-ray Diffraction (XRD) of W-Doped VO_2_ Thin Films

**Figure 10:**
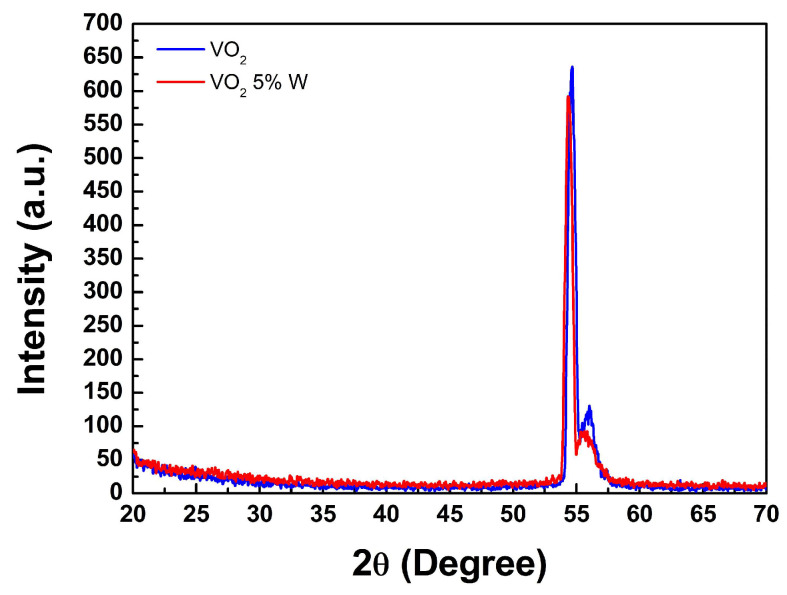
X-ray diffraction (XRD) pattern of undoped VO_2_ and 5% W-doped VO_2_ thin films.

X-ray diffraction (XRD) pattern analysis of the prepared thin films was performed using a high-resolution multifunctional X-ray diffractometer (XRD) system. The basic diffraction principle of Bragg’s law was employed. Bragg’s law states that when X-rays with a fixed wavelength are directed at a sample and the angle θ between the incident X-ray beam and a crystal plane satisfies the condition 2dsinθ = mλ, where d is the spacing between crystal planes, λ is the wavelength, and m is an integer, as diffraction occurs. Each compound or element has a unique X-ray diffraction spectrum, and it can be identified by searching the JCPDS database. The grain size of VO_2_ thin film can be considered an important parameter for controlling the material’s transition temperature [30]. XRD characterized the VO_2_ thin film samples in this study for two main reasons: firstly, to verify whether monoclinic VO_2_ was obtained, and secondly, to be able to quantitatively estimate the grain size based on the Scherrer equation (or qualitative estimation was achieved through FE-SEM). In Figure 10, the X-ray diffraction patterns for the undoped VO_2_ and 5% W-doped VO_2_ thin films are presented, respectively. According to the literature, the appearance of the VO_2_ (220) crystalline phase is expected at a diffraction angle of approximately 55 degrees [31]. In our experimental results, the undoped VO_2_ film has a sharp diffraction peak at 54.7°; while the VO_2_ doped with 5% tungsten exhibits a diffraction peak near 54.3°. As the W doping content increases, the peak value of the undoped VO_2_ film slightly shifts to a lower angle. The incorporation of W^6+^ into VO_2_ results in a shift of the (220) peak, which slightly shifts to a lower angle as the W content increases [32]. It can be inferred that our measurement result is consistent with the literature.

#### 3.3.2. Surface Morphology of Undoped VO_2_ and W-Doped VO_2_ Thin Films

**Figure 11:**
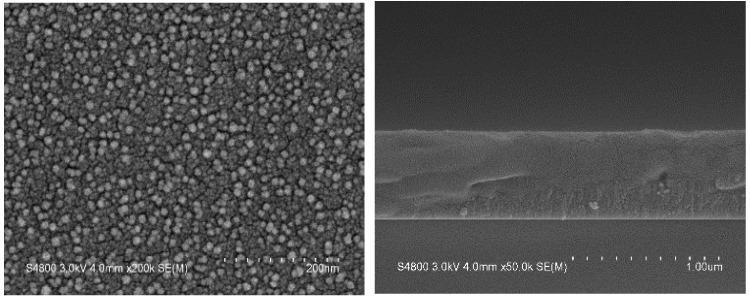
SEM images of undoped VO_2_ thin films deposited on Si substrate: (**Left**) Top–down view, (**Right**) Cross–sectional view.

**Figure 12:**
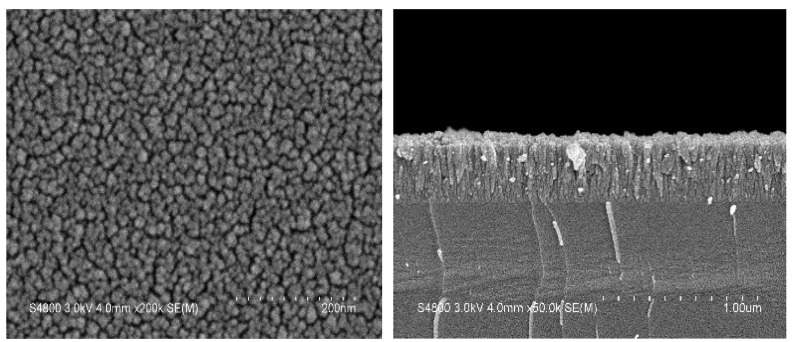
SEM images of 5% W-doped VO_2_ thin film deposited on Si substrate: (**Left**) Top–down view, (**Right**) Cross–sectional view.

A high-resolution field emission scanning electron microscope (FE-SEM) was used to examine the surface morphology and cross-sections of the VO_2_ thin films. The film’s microstructure analysis is based on scanning electron microscope (SEM) measurements of cross-sectional and top–down views of vanadium dioxide thin films deposited on Si substrate. Figure 11 and Figure 12 show the SEM images of undoped VO_2_ and 5% W-doped VO_2_ thin films. With the addition of tungsten doping, larger and more densely dispersed particles are observed on the surface of the thin films. In contrast, undoped VO_2_ thin films exhibit smaller particles. Compared with vanadium, tungsten has a larger ionic radius, which affects the stability and activity of grain boundaries, making them easier to move. This results in the potential migration and coalescence of existing grains at the grain boundaries and leads to the enlargement of crystal grains in the vanadium dioxide thin films.

## 4. Conclusions

This study employed an electron gun evaporation combined with an ion-assisted deposition technique to fabricate tungsten-doped vanadium dioxide thin films with doping contents of 3%, 4%, and 5%, as well as undoped VO_2_ thin films. All deposited VO_2_ thin films have a thickness of about 100 nm. This investigation revealed that higher W doping contents resulted in lower transmittance in the visible light spectrum (400–750 nm) and the infrared spectrum (2.5 μm to 5.5 μm). The various W doping VO_2_ thin films have been explored to not only evaluate film quality but also to understand doping effects. Along with the phase transition, the optical and electrical properties of VO_2_ films undergo significant changes. The refractive index of VO_2_ thin films increases slightly with the increase of tungsten doping concentration. As the heating temperature increases, the resistivity of the VO_2_ thin film gradually decreases with the increase of W doping amount. In this work, as the W doping ratio increases, the residual stress in the VO_2_ thin film gradually decreases, and the surface roughness increases. The VO_2_ thin film heating experiment showed that compared with other doped VO_2_ films and undoped thin films, the transmittance of 5% W-doped VO_2_ films decreased by about 11%. Meanwhile, the thermal expansion coefficient of the vanadium dioxide thin film was determined to be 5.1 × 10^−6^ °C^−1^, and the biaxial modulus was found to be 396 GPa. Raman spectroscopy was used to characterize the thin film samples. Raman spectral peaks of the W-doped VO_2_ films and undoped VO_2_ thin films are consistent with the literature [33]. All four VO_2_ thin films feature a signature Raman peak at 612 cm^−1^. The undoped VO_2_ film has a sharp diffraction peak at 54.7°; while the VO_2_ doped with 5% tungsten exhibits a diffraction peak near 54.3°. As the W doping content increases, the peak value of the undoped VO_2_ film shifts slightly to a lower angle. Future work will involve scaling up W-doped VO_2_ thin film deposition on larger substrates and optimizing process parameters for potential applications in smart glass windows, tunable optical filters, and optical switches.

## Data Availability

The data are not publicly available due to privacy.
